# Low-temperature direct bonding of silicon nitride to glass

**DOI:** 10.1039/c7ra08854j

**Published:** 2018-01-09

**Authors:** Limor Pasternak, Yaron Paz

**Affiliations:** Department of Chemical Engineering, Technion Israel paz@tx.technion.ac.il

## Abstract

Direct bonding may provide a cheap and reliable alternative to the use of adhesives. While direct bonding of two silicon surfaces is well documented, not much is known about direct bonding between silicon nitride and glass. This is unfortunate since silicon nitride is extensively used as an anti-reflection coating in the PV industry, often in contact with a shielding layer made of glass. A series of bonding experiments between glass and SiN was performed. The highest bonding quality, manifested by the highest bonding energy and lowest void area, was obtained with pairs that had been activated by nitrogen plasma followed by post-contact thermal annealing at 400 °C. HRTEM imaging, HRTEM-EDS and EELS measurements performed on the thin films prepared from bonded samples by Focused Ion Beam (FIB) revealed a clear defect-free interface between the silicon nitride and the glass, 4 nm in thickness. ATR FT-IR measurements performed on activated surfaces prior to contact indicated the formation of silanol groups on the activated glass surface and a thin oxide layer on the silicon nitride. An increase in the bearing ratio of the glass following activation was noticed by AFM. A mechanism for bonding silicon nitride and glass is suggested, based on generation of silanol groups on the glass surface and on oxidation of the silicon nitride surface. The results point out the importance of exposure to air, following activation and prior to bringing the two surfaces into contact.

## Abstract 2

Suggested mechanism for direct bonding of SiN to glass: (A) raw materials, (B) surfaces after plasma activation and exposure to air, (C) surfaces in contact, (D) formation of water molecules, (E) covalent bond formation and water removal.

## Graphical abstract

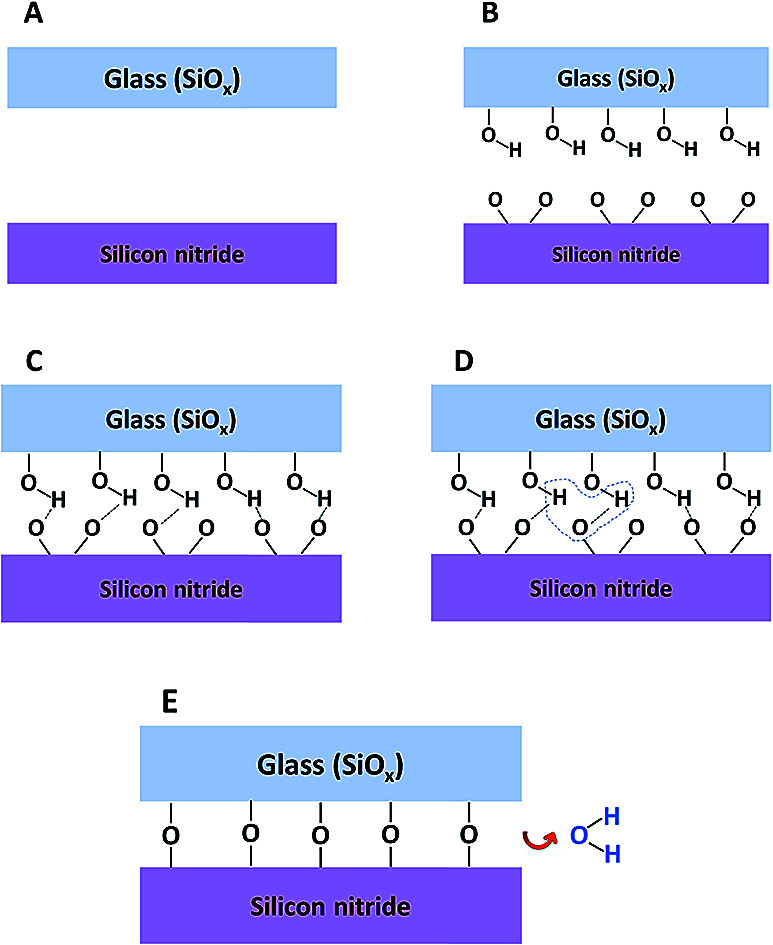

## Introduction

Over recent years, the direct bonding of wafers has become an important technology used for the integration of materials in microelectromechanical systems (MEMS),^1^ microelectronics,^2^ optoelectronics,^3^ vacuum packaging,^4^ hermetic sealing^5^ and encapsulation.^6^ Direct bonding is based on bringing together two ultra-flat, smooth, clean surfaces, thus forming many weak interactions (van der Waals forces, hydrogen bonding, capillary forces or electrostatic forces) that hold together the two surfaces. This is followed by an annealing step at elevated temperatures aimed at converting the weak physical interactions into strong, covalent bonds. The technique was mostly studied for bonding silicon to silicon. Here, two different chemistries are possible: hydrophobic bonding and hydrophilic bonding. Hydrophilic bonding is performed on oxide/hydroxide-terminated wafers, whereas in hydrophobic wafer bonding the surfaces to be bonded are hydrogen terminated (Si–H_2_ and Si–H), following oxide etch by hydrofluoric acid. At room temperature, the interactions formed in the hydrophobic bonding process are weaker than the interactions formed in the hydrophilic bonding process, since the former rely on vdW interactions, whereas in the latter hydrogen bonds are formed. Annealing the contacted hydrophobic surfaces at 300–700 °C generates strong Si–Si covalent bonds, while emitting hydrogen molecules.^7^ The lack of any intermediate layer makes this type of bonding ideal in cases where epitaxial properties are important, such as in p–n junctions.

Unlike hydrophobic bonding of silicon, hydrophilic bonding involves a thin intermediate layer made of silicon dioxide. According to a widely-accepted mechanism for hydrophilic wafer bonding between SiO_2_ and SiO_2_,^8^ the first step comprises of formation of hydrogen bonds between the hydroxyl-terminated surfaces. Here, the presence of mediating water molecules may assist in overcoming sub nanometer roughness, which impedes direct hydrogen-bonding. In the next stage, a condensation reaction occurs, yielding Si–O–Si bonds between the surfaces while releasing water. An annealing step is required to drive the water molecules away from the interface. If both bonded substrates are made of the same material, one may achieve a bond-strength equal to that of the bulk material,^9^*i.e.* higher than 1 J m^−2^.^10^ The water molecules may diffuse along the bonded interlayer (a relatively slow process) or into the oxide layer or, alternatively, they may react with silicon to form silicon dioxide and hydrogen.

Activating the surfaces of the wafers prior to contact by plasma treatment may induce sputtering effects as a result of ion bombardment,^11^ but at the same time, may increase the concentration of surface hydroxyls following surface reaction with atomic oxygen and adsorbed water.^12^ Generally speaking, plasma activation strengthens the bonding at the pre-annealing stage, alleviates the requirements for surface smoothness, and may reduce the required temperature in the post-contact thermal annealing stage. In the case of oxygen plasma, claims were made that the formed oxide following oxygen plasma activation originated from oxygen ions.^13^ The outcome is a charged microenvironment that may increase the diffusivity of species (water and hydrogen) at the interface.^13,14^

Intimate contact between glass and silicon nitride is important in silicon solar cells, which are still the working horses of the PV industry. Here, a thin layer of silicon nitride serving as an antireflection coatings is protected by a glass covering.^15^ While direct bonding between silicon and silicon oxide is well-documented, there is hardly any documentation on low temperature plasma activated direct bonding between silicon nitride and glass. In what follows, high quality direct bonding between silicon nitride and glass is reported. Of specific interest is the ability to perform the process at low temperatures, which are commensurate with the conditions and processes used for manufacturing of silicon solar cells.

## Experimental

### Sample preparation

The silicon nitride surfaces used for this study comprised of commercially available double-sided polished silicon wafers (Cz, 〈100〉, P-type, *D* = 100 mm, 500 μm in thickness) onto which an amorphous layer, 0.2 μm in thickness, of silicon nitride, denoted here as SiN, was deposited by LPCVD on both sides (Siltronix Silicon Technologies). The glass wafers used for this study were 500 μm thick Borofloat33 glass wafers (SCHOTT). All wafers had very low roughness (under 0.5 nm), low bow value (<30 μm) and low thickness variation (<5 μm). The first step in the preparation of samples comprised of 10 minutes immersion of the two types of wafers in an SC1 solution (H_2_O : H_2_O_2_ : NH_4_OH, 7 : 2 : 1 by vol) at 75 °C. The wafers were then exposed to plasma (either oxygen or nitrogen) in a commercial chamber (EVG810LT, EV-Group) under specific conditions, as detailed below. Following plasma activation of both surfaces, the wafers were cleaned by a megasonic DI water cleaner (EVG301, EV-Group) to remove particles residing on the surfaces and then were brought into contact (force: 2 kN) in a wafer bonder (EVG501, EV-Group). This was followed by an annealing step (300–400 °C as specified below) performed consecutively in the same wafer bonder. Process parameters that were investigated included activation time and pressure, temperature and duration of annealing. The gas flow, the megasonic cleaning parameters (20 W, 1 min), and the applied force during bonding (2000 N, 2 h) were held constant for all experiments. The SC1 cleaning, the megasonic cleaning, the activation and bonding procedure were performed in a class 100 clean-room. All bonded wafers were characterized, tested and inspected to estimate the bond quality.

The large number of process parameters and the possibility of cross-over effects of these parameters could have required a very large set of prepared samples, whose preparation and characterization could have been beyond the time constrains of this study. To overcome this problem, a Design of Experiments (DOE) technique (JMP, SAS Ltd.) was applied, utilizing previous experience in Si–Si plasma assisted direct bonding processes to yield the minimal number of required experiments (8), whose parameters are given in Table 1.

**Table 1:** The set of samples used for this study based on DOE analysis

Sample type	Gas	Activation gas pressure [mbar]	Activation time [s]	Annealing temp [°C]	Annealing time [h]
1	N_2_	0.3	20	300	1
2	O_2_	0.7	80	400	1
3	O_2_	0.7	20	300	2
4	N_2_	0.3	80	400	2
5	N_2_	0.7	80	300	2
6	O_2_	0.3	20	400	2
7	O_2_	0.3	80	300	1
8	N_2_	0.7	20	400	1

### Characterization

The characterization of the samples comprised of surface characterization prior to bonding (but after activation) and studies on the chemical, structural, and mechanical (bond strength) properties of the glass–SiN bonded samples. Evaluating the integrity of the bonding was achieved easily by visual inspection of voids formed between the glass and the SiN, and using their number and total area as indicators for the quality of bonding. These indicators were compared with mechanical properties measured by the crack opening method^16^ and by shear stress measurements. The shear tests provided two kinds of results; one was the force needed to induce failure of the bonded pair and the other was the failure type: cohesive or adhesive. The failure type was easily observed by optical microscopy. The shear tests were performed in Royce650-Universal bond tester machine using 3 mm × 3 mm dies. For the crack opening method, a razor blade was used in a manual manner. The lengths of the cracks were then used to calculate the bonding energies.

A Focused Ion Beam (FIB-STRATA 400S, FEI) technique was used to detach a small, thin specimen from the bonded area. This specimen was then imaged by a high resolution TEM (Titan, FEI). Results were corroborated by electron energy loss spectroscopy (EELS) performed on the same specimen.

A variety of techniques were used in order to study the surfaces prior to bonding. These techniques included XPS (Thermo-VG, SIGMA probe), ATR-FTIR (Vertex 70V, Bruker), AFM (DI-3100, Digital Instruments) operating in tapping mode and goniometry with water (Z500 Goniometer, Rame-hart). Several samples were coated with a hydrophobic adhesion promotor (HMDS, YES-310TA, Yield Engineering Systems) as an indicative tool. Care was made to shorten the air-exposure time between plasma activation and measurements (5–30 min, depending on technique).

## Results and discussion

### Macroscopic characterization of bonding

#### Macroscopic characterization of SiN–glass bonding

**Fig. 1:**
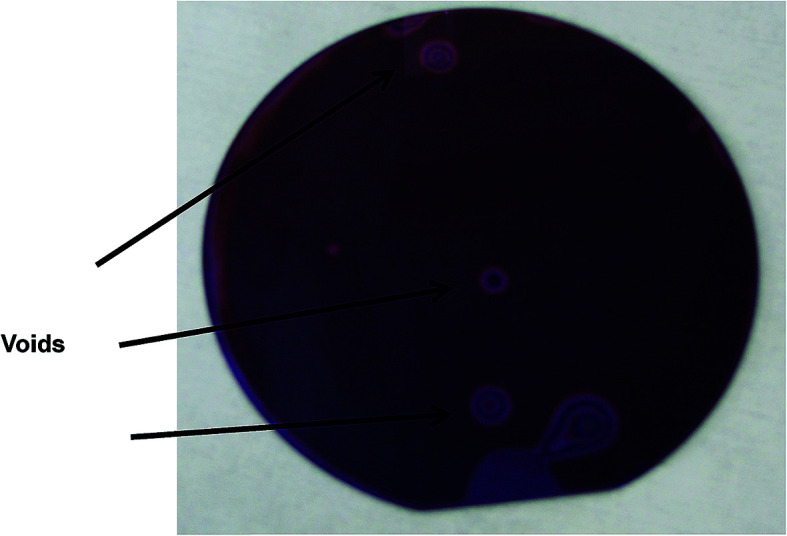
A SiN–glass bonded wafer. The voids are easily observed by their Newton rings.

Fig. 1 presents an example of a silicon nitride-coated silicon wafer, directly bonded to a borosilicate glass wafer. The lower surface is opaque (SiN on Si), whereas the upper is made of a transparent glass, enabling to observe bonding defects. The figure reveals that most of the area is bonded, still there are several easily observed non-bonded areas between the two wafers.

Table 2 presents the mechanical properties of the various types of samples. These include the number of voids per wafer, the total area of the voids per wafer, the bonding energy, the shear load and the types of failure (*i.e.* cohesive or adhesive) during the shear test. The observation of a cohesive failure, represented by the formation of two rough surfaces, is in particular important as it indicates that the bonding was stronger than the strength of the bonded materials. In all cases of cohesive failures, the failures occurred on the glass side.

**Table 2:** The mechanical properties of the various types of samples

Sample type	Failure type in shear test (C = cohesive), (A = adhesive)	Shear load [kgF]	Number of voids per wafer	Total area of voids per wafer (cm^2^)	Bonding energy [J m^−2^]
1	C and A	24 ± 8	8 ± 3	2.5 ± 0.8	0.3 ± 0.2
2	C and A	42 ± 2	4 ± 3	2.5 ± 0.8	0.6 ± 0.5
3	C and A	32 ± 21	7 ± 6	2.1 ± 1.8	0.1 ± 0.05
4	C	18 ± 3	3 ± 1	0.3 ± 0.2	0.6 ± 0.1
5	A	12 ± 7	43 ± 36	25 ± 15	0.02 ± 0.02
6	A	3.2 ± 1.8	7 ± 1	2.3 ± 0.7	0.002 ± 0.001
7	C and A	13 ± 7	2 ± 1	1.8 ± 1.7	1.1 ± 0.01
8	C	20 ± 7	1 ± 1	0.008 ± 0.001	0.3 ± 0.2

Of the various types of samples only type 4 and type 8 always yielded cohesive failures during the shear tests. Both types of samples had no more than 1–3 voids per wafer and less than 0.5 cm^2^ of void area per wafer. They revealed also a high shear load (app. 20 kgF) and high bonding energy (0.6 and 0.3 J m^−2^, respectively). These values are in particular interesting as, unlike Si–Si bonding, they represent bonding strength between two materials that are quite different. It is noteworthy that during performing the crack opening test, a local breakage of the wafer's edge was observed in many attempts, reflecting the high bonding strength obtained during bonding, causing the glass to be the weakest link. As shown in Table 1, these two types were prepared by activation with nitrogen plasma followed by thermal annealing at 400 °C.

In contrast, samples type 5 and 6 revealed the lowest quality of bonding: adhesive failure type during shear test measurements, low bonding energies and large number of voids per wafer. The qualities of samples types 1, 2, 3 and 7 varied significantly from wafer to wafer. This is well manifested by the large standard deviation in the mechanical properties between wafers. At the same time, the deviation between measured points at the same wafer (at least five points) were very small. The large STD between wafers may suggest the existence of another, unknown, parameter. A post-consideration of the preparation process yielded that in all cases the time between activation and contacting was kept constant (five minutes). Likewise, analyzing the cleaning procedure for possible variations did not indicate any differences in the cleaning procedure. Since each sample was prepared at a different date, the variance could be connected to variations in the external conditions in the clean room. Otherwise, the missing parameter might be related to some deviations in the properties of the raw material. As mentioned before, the failure type was found to be a good representative of the bonding quality: wafers that failed cohesively showed always higher shear load values, higher bonding energies and smaller number of voids than wafers that failed adhesively in the shear test measurements.

Measurements on set of wafers that had been bonded at a variety of process parameters revealed that altering the gas pressure during activation (0.3–0.7 mbar), and altering the activation time (20–80 s) had very small effect (if at all) on the quality of bonding, in terms of bonding energies, shear strength, number of voids and shear test failure type. In contrast, using nitrogen for activation produced higher-quality bonding than using oxygen for activation. Likewise, an annealing temperature of 400 °C was found to yield higher-quality bonding than an annealing temperature of 300 °C. These findings are quite similar to previous reports on Si–Si direct bonding.^17^

Examining the bond energy *versus* voids area per wafer revealed that high bond energies (0.1–1.1 mJ m^−2^) may be achieved only when the total voids area per wafer was lower than 4 cm^2^. In a similar manner, it was found that the bonding energy may be high only if the number of voids is below a critical number per wafer (<6). It is noteworthy that, to our surprise, plotting the shear strength *versus* the bond energy for the various samples did not indicate a clear correlation between the two parameters, probably due to the different failure mechanisms.

In order to further study the bonding between SiN and glass, a comparison was made with direct bonding of SiN to SiN. For that, the same procedure and conditions that yielded the best quality SiN–glass bonding (type 4 process) was used. Unlike SiN–glass bonding, the bonding between the two SiN wafers was found to be very weak. This was manifested by a large number of macroscopic voids (as viewed by IR imaging) and by the separation of the bonded wafers during dicing, thus preventing the performing of shear test measurements.

### Microscopic characterization of bonding

**Fig. 2:**
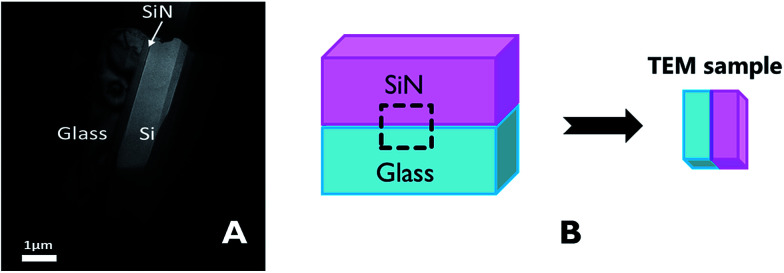
(A) Location of the TEM sample on the bonded wafers, (B) TEM sample made by FIB.

The characteristics of the glass–SiN direct bonding was studied also by a variety of microscopic techniques. For this, thin films of the bonding area were prepared by the Focused Ion Beam (FIB) method. The FIB detached a thin sample from the interface of the bonded wafers (Fig. 2), thus enabling a side view characterization of the bonded area. Upon preparation, the films were studied by TEM, EDS, and EELS.

**Fig. 3:**
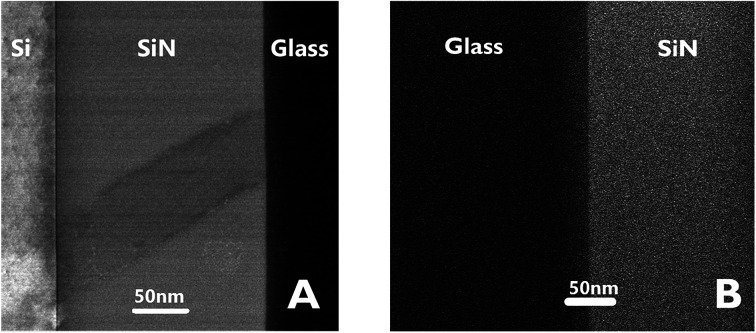
STEM images of the interfaces between Si–SiN–glass.

Fig. 3 presents STEM images of the bonded area. The images reveal a smooth interface between the SiN and the glass compared to the interface between the Si and the deposited SiN above it, where a clear thin dark line, 5 nm in thickness, appears between the two materials.

**Fig. 4:**
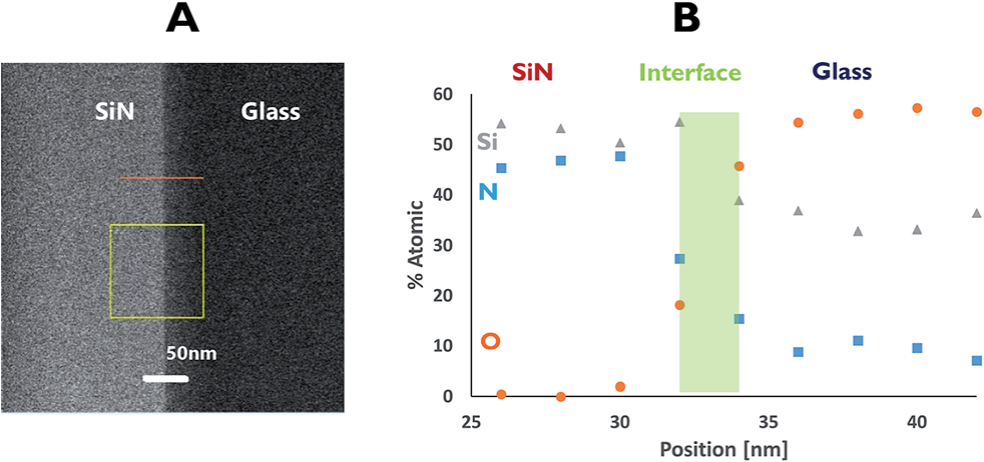
EDS profile for the SiN–glass interface.

EDS was used to understand the nature of the interface area. Here, measurements across the bonding line were taken every 2 nm with a probe size diameter of 1 nm. The EDS analysis (Fig. 4) revealed an interface of approximately 4 nm in length, based on changes in the atomic percentage of oxygen as the point of data collection moved from the glass (∼60% of oxygen) to the SiN (originally no oxygen). This thickness was corroborated by following the increase in the atomic percentage of nitrogen. At the centre of the interface an atomic ratio of 1 : 1.3 : 2.2 between silicon, oxygen and nitrogen, respectively, was measured.

**Fig. 5:**
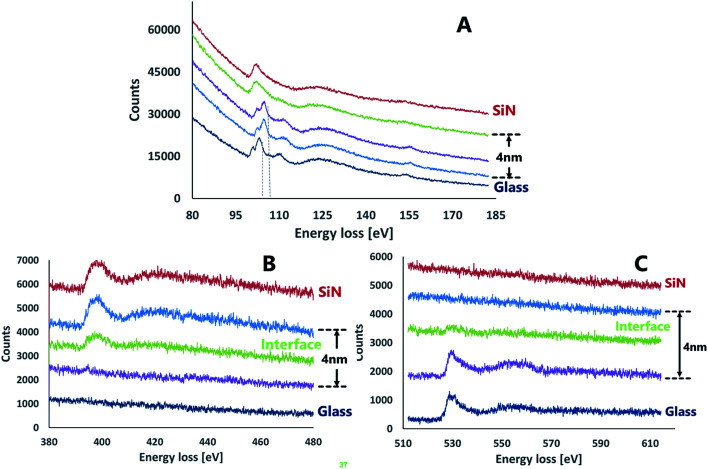
EELS spectra of the interface area between SiN and glass. (A) Si-L_2,3_ (B) N-K (C) O-K.

*In situ* EELS measurements across the SiN–glass region were performed to study the chemical modification following the bonding and to examine the interface between the wafers. The EELS measurements were performed in three energy ranges corresponding to characteristic energy losses for silicon, oxygen and nitrogen. The Si-L_2,3_ energy loss spectra measured for SiN and glass (Fig. 5A) were shifted by 4–6 eV relative to data reported for Si_3_N_4_ and amorphous silica (according to ref. 18–20). This consistent difference is probably due to different stoichiometric proportions within the nitride. Besides these variations, the Si-L_2,3_ EELS spectra contained all the expected features and the peak intensity ratios were similar to previously reported. The EELS spectra for the silicon region were consistent with the EDS results, showing a very thin interface layer between the SiN and the glass. An extremely sharp transition between two successive EELS measurements in the interface region, 2 nm apart, were observed. Both spectra resembled the bulk spectra of the nitride and the glass, nonetheless, there was a small shift (3.3–3.7 eV) between the bulk glass spectrum and that of the interfacial region. This shift may indicate the existence of silicon oxy-nitride at the interface.^21^ Formation of silicon oxynitride is thermodynamically possible as the Gibbs free energy for the formation of silicon oxynitride from silica and silicon nitride is negative (−111.8 kJ mole (Si_2_N_2_O)^−1^).^22,23^

The nitrogen energy loss spectra (Fig. 5B) showed a clear interface that contained an attenuated peak relative to SiN bulk. The energy loss values for the nitrogen showed repeatedly a difference of ∼6 eV from the theoretical data for silicon nitride. Energy loss spectra of oxygen (Fig. 5C) also exhibited the existence of an interface between the layers.

The data collected from both EDS and EELS suggest the existence of a 2–4 nm interface formed between the bonded wafers which contains Si, O and N in various ratios. Coupled with the data from the Si-L_2,3_ EELS measurements, this observation may indicate the presence of a silicon oxynitride compound in the interfacial region.

### Study of plasma-activated surfaces

**Fig. 6:**
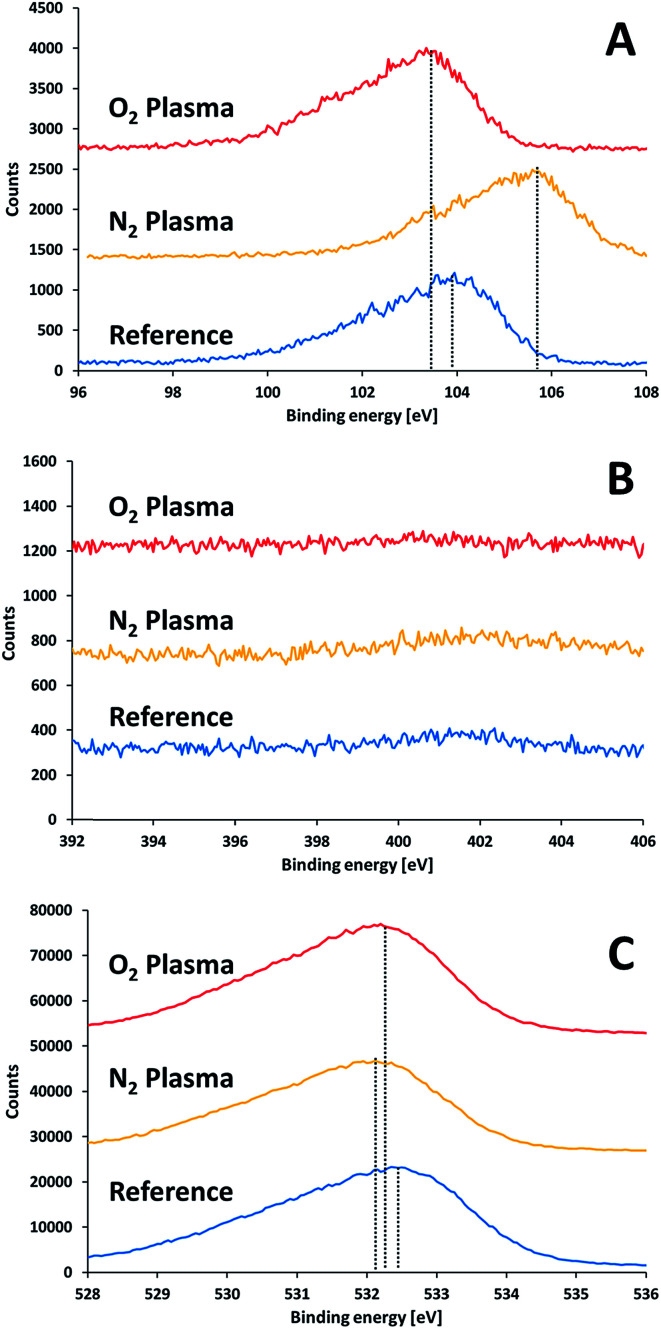
XPS results for glass substrate prior to and after plasma treatments: (A) silicon 2p, (B) nitrogen 1s, (C) oxygen 1s.

Surface characterization measurements following activation but prior to bonding were performed to study the effect of the plasma activation treatment on the surface and its relation to the bond strength. Fig. 6 presents XPS measurements performed on the surface of SiN and glass wafers following exposure to different plasma treatments. Here, the samples were exposed to air for 30 minutes after activation prior to introduction into the XPS vacuum chamber. Eliminating charging effects was performed by subtracting the difference between the measured position of the C1s signal and the standard value of C1s (284.8 eV) from the obtained measurements. In general, silicon nitride and glass samples were charged by 5–8 V and 63–70 V, respectively. It is noteworthy that such shifts due to charging (and even higher) have been reported for similar systems.^24^ Covering part of the sample with a copper grid did not reduce the charging of the glass significantly.

The XPS results for non-activated and activated surfaces of glass (Fig. 6B) did not reveal any nitrogen on the glass surfaces, *i.e.* the absence of any oxynitride species, regardless of the type of activating gas (oxygen/nitrogen). This proves that the oxynitride found at the interface between the glass and the SiN in the bonded samples originated neither from nitrogen in air, nor, in the case of activation by nitrogen, from nitrogen species in the nitrogen plasma.

The Si2p XPS peak in the glass (Fig. 6A) revealed a slight shift to lower binding energy upon activating with oxygen plasma (from 103.8 eV to 103.4 eV). Such a shift could be due to formation of silanol (Si–OH) groups on the surface. Contrary to the effect of oxygen plasma, activating of glass by nitrogen plasma shifted the Si2p peak into a higher binding energy (from 103.8 eV to 105.6 eV). The shift to very high binding energy was quite peculiar since the expectation was the opposite: the binding energy of 2p electron in a silicon oxynitride is lower than 103.8 eV and as low as 102 eV for silicon nitride. Therefore, one should look for an alternative explanation for the mysterious shift. The strong upward shifting indicated the presence of a specie of a very high electronegativity nature. Such a shift could only be explained by the presence of fluorine, since the formation of SiO_*x*_F_4−*x*_ can shift the silicon peak to similar energies.^25^ Indeed, a survey for the presence of fluorine revealed the existence of a fluorine peak at ∼685 eV for the nitrogen-activated glass surface. Fluorine was found also in all activated samples (but not in the non-activated), however its effect on the binding energies of the other samples was minute. Therefore, its source was in the activation process. The fact that fluorine was present regardless of the type of activation gas suggests that its source was the lift pins of the sample holder, which were made of Teflon. The binding energy of oxygen did not shift by much upon activating. It revealed a weak shift towards lower energies. This is in accordance with the formation of silanols. The oxygen to silicon ratio in glass prior to activation was slightly higher than 2 (2.24). This ratio was slightly increased upon activation (2.35 and 2.51 following activation with oxygen and nitrogen plasma, respectively). The higher ratio obtained upon activation with nitrogen suggests (albeit does not prove) that, under the experimental conditions, the nitrogen plasma provided better local environment for surface oxygenation, upon exposure to air, than activation with oxygen.

**Fig. 7:**
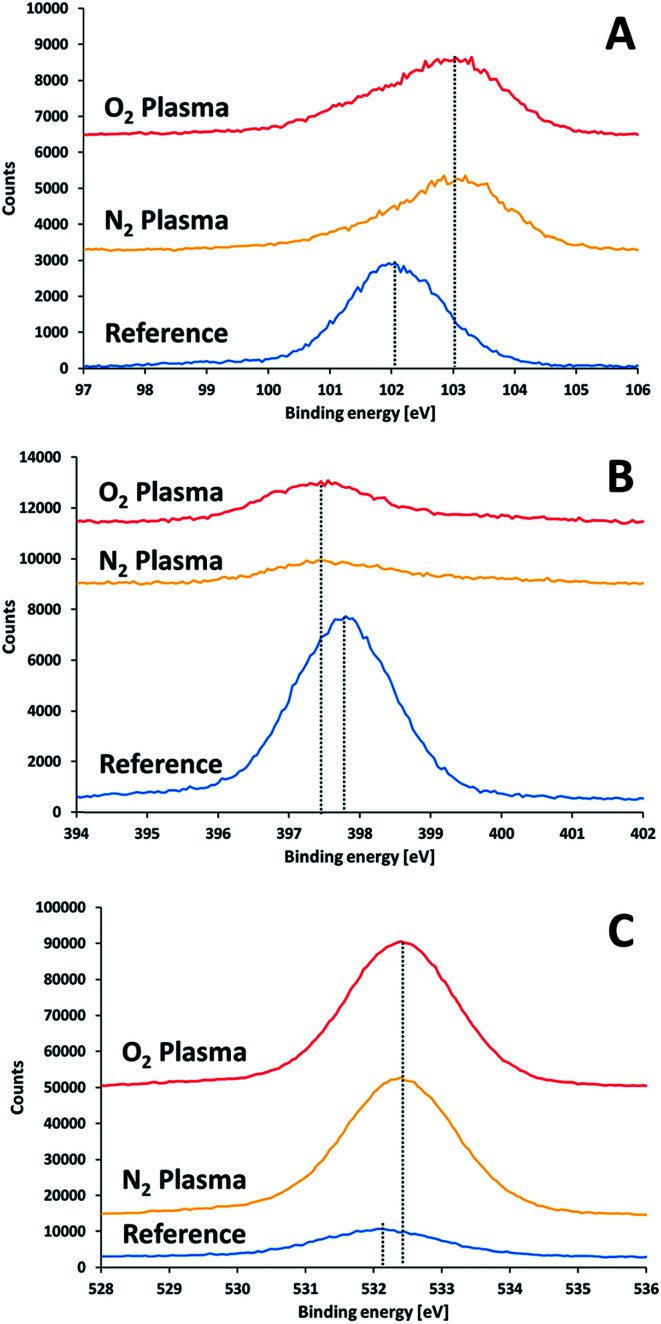
XPS results for a SiN substrate prior to and right after plasma treatment: (A) silicon 2p, (B) nitrogen 1s, (C) oxygen 1s.

XPS results of non-activated and activated SiN substrates showed differences in the positions of the Si2p, N1s and O1s peaks (Fig. 7). Upon surface activation, a shift in the Si2p peak from 102.1 eV to 103.1 eV was found. The extent of this shift did not depend on the type of activation gas (nitrogen or oxygen). This shift to higher binding energies implies the formation of a polar oxide layer, where the interactions of the silicon electrons with their nuclei is stronger that in the less polar SiN. Reconstruction of Si peak from the two peaks (103.1 eV and 102.1 eV) suggested a SiO_*x*_ : SiN ratio of 1.25 for the two activation gases.

The activated surfaces of the silicon nitride demonstrated significant changes in the atomic ratio following activation (Table 3). The most notable change was an increase in the ratio between oxygen to silicon, from 0.34 to 1.79 and 1.92 upon activation with oxygen plasma and nitrogen plasma, respectively. This increase in the O : Si ratio, manifest the tendency of the activated surface to be oxidized upon exposure to air.

**Table 3:** Atomic concentrations (%) of Si, O, N prior to and after activation

	Glass			SiN		
	Si	O	N	Si	O	N
Prior to activation	30.7	68.7	0.6	40.2	13.6	46.2
Following activation with oxygen plasma	29.6	69.5	0.9	31.6	56.7	11.7
Following activation with nitrogen plasma	28.2	70.9	0.9	31.1	59.8	9.0

A very interesting finding is the decrease in the N : Si ratio upon activating the surface. If the only effect of plasma activation was to introduce oxygen upon exposing to air, one could have expected that the ratio between nitrogen to silicon (app. 1.15 prior to activation) would remained after activation. The fact that this ratio decreased significantly (from 1.15 down to 0.3) suggests that nitrogen species were removed from the surface due to activation, and are replaced by oxygen upon exposure to air, thus forming a (sub-)oxide on the surface, which may participate later in formation an array of covalent bonds with the activated glass. It should be noted that the N : Si ratio was higher in the oxygen-activated surface (0.37) than in the nitrogen-activated surface (0.29). This is in correlation with the higher O : Si ratio obtained upon activating with nitrogen.

The claim for a depletion of nitrogen due to activation is supported also by the shift in the binding energy of N1s towards lower energies observed upon activation, from 397.9 eV to 397.4 eV, since such a shift was reported before in an XPS study performed on a series of SiN_*x*_ compounds.^37^ No evidence for Si_2_=N–O structure was found, as such bond structure should have been expressed by a spectral peak at a binding energy of 401.0 eV.^38^ Hence, it can be concluded that plasma activation partially replaces nitrogen with oxygen and that the oxygen is attached to the silicon atoms only.

**Fig. 8:**
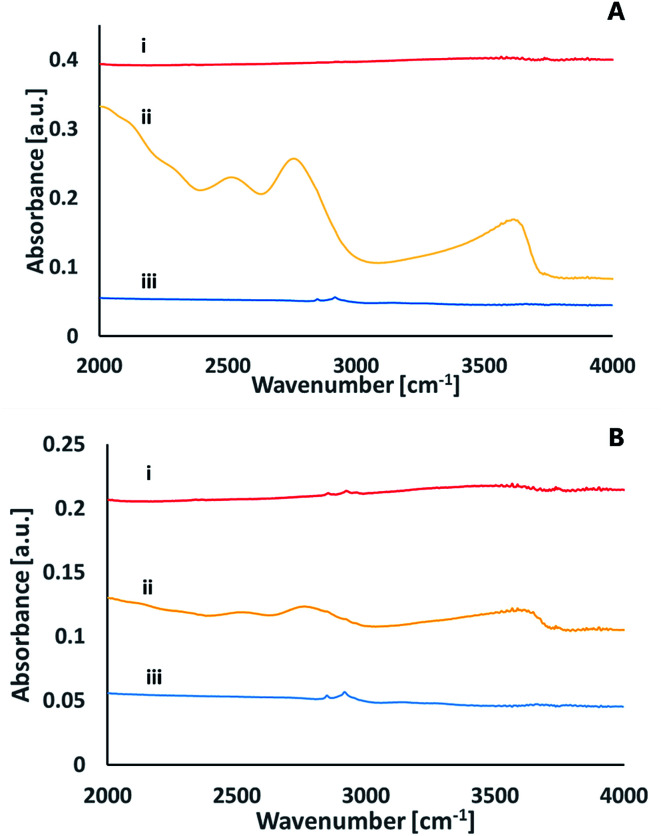
ATR FT-IR spectra of a glass surface following activation with nitrogen plasma (A) or with oxygen plasma (B), prior to activation (i), right after activation (ii) and 24 hours after activation (iii).

To better understand the chemical species formed on the surfaces upon activation, infrared spectroscopy was utilized in the Attenuated Total Reflection (ATR) mode. Fig. 8 presents the ATR spectra of a glass surface prior to activation, right after activation and 24 hours later. The data was recorded upon activation with nitrogen plasma (Fig. 8A) as well as with oxygen plasma (Fig. 8B). While no significant peaks were observed for the non-activated samples (except for some C–H stretch transitions at 2900–3000 cm^−1^ due to organic contamination), distinct peaks were observed right after excitation. These peaks at ≈3630 cm^−1^, ≈2770 cm^−1^, ≈2520 cm^−1^, ≈2050 cm^−1^ eventually disappeared within 24 hours of exposure to air. The 3630 cm^−1^ peak can be assigned to Si–OH stretching mode,^26^ the 2770 cm^−1^ peak relates to the stretching mode of hydrogen-bonded (Si)O–H group,^27^ the 2520 cm^−1^ peak is likely to represent a stretching mode of (Si)O–H group in another site that correlates with weaker hydrogen bonding with certain non-bridging oxygens of a glass matrix^27^ and the small peak at 2050 cm^−1^ represents multi-phonon vibrations of the glass matrix.^27^

The ATR-FTIR measurements thus reveal that upon excitation and short exposure to air, silanol groups are formed on the surface of the glass. The surface concentration of the silanols eventually decreases over time, in correlation with a loss in the ability for direct bonding after prolonged exposure to air. This decrease could be due to formation of the less active surface Si–O–Si, partially covered with some organic contamination.

A comparison between the ATR-FTIR spectra of glass samples activated by nitrogen and ATR-FTIR spectra of glass samples activated by oxygen plasma reveals similar features for the two types of surfaces. Yet, the intensity of the peaks upon activation with nitrogen was significantly higher than that observed upon activation with oxygen. While it is true that comparing signal intensities between different samples should be taken with large precaution, the difference was so significant to allow suggesting that nitrogen activation yields more silanols than activation with oxygen. This conclusion is in correlation with the XPS results (see above).

**Fig. 9:**
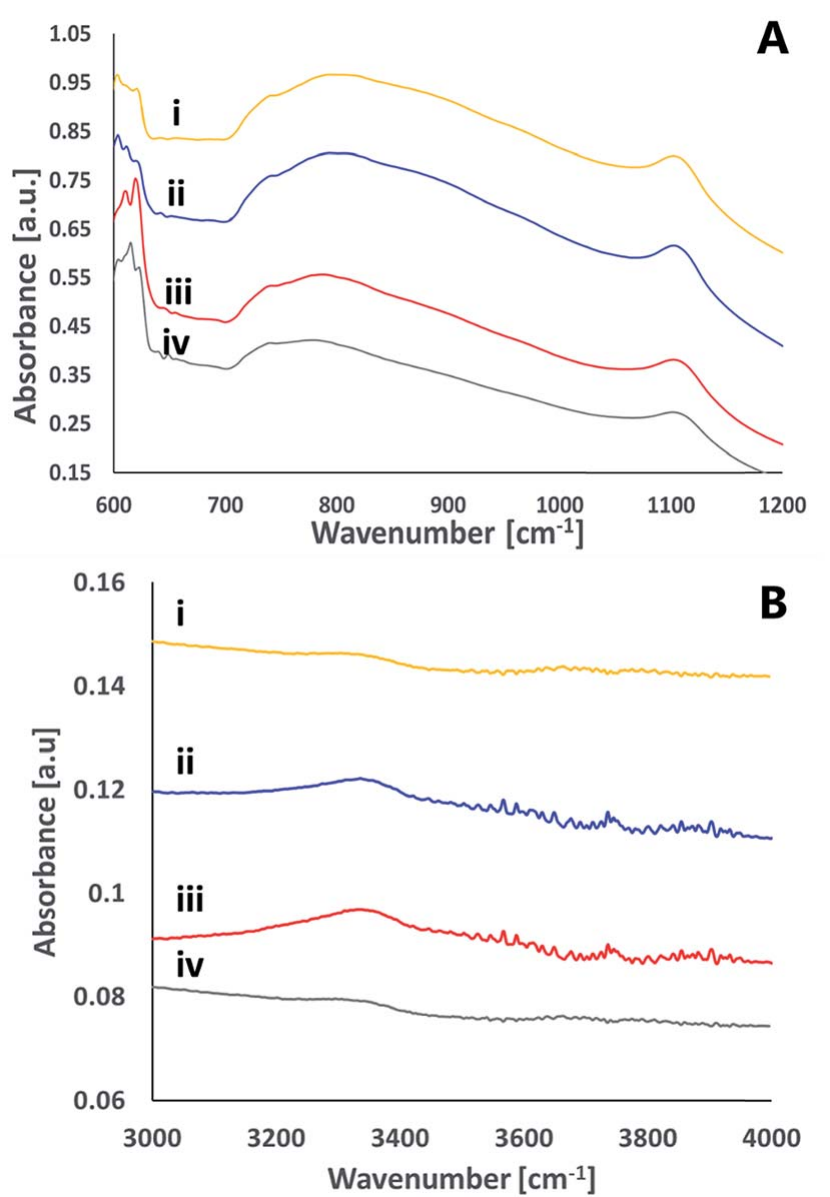
ATR FTIR spectra of a SiN surface. (i) Prior to activation (ii) immediately after activation by nitrogen plasma (iii) immediately after activation by oxygen plasma (iv) 24 hours after activation by oxygen plasma.

ATR FT-IR measurements were utilized also to study the effect of activation on the surface of the silicon nitride wafers (Fig. 9). The spectra was dominated by a broad asymmetric and symmetric stretch Si–N peaks at 800 cm^−1^ and at 1100 cm^−1^, respectively.^28^ These peaks were not altered upon activation. In addition to these peaks, the figure reveals several small peaks that exist only in the activated spectra (regardless of the activating gas) that disappear within 24 hours after activation. Among these peaks is a peak at ≈3340 cm^−1^ often assigned as N–H stretching.^29^ A very weak peak at ≈3760 cm^−1^ was revealed in both oxygen and nitrogen-activated surfaces, which could indicate some Si–OH moieties.^30^ The peaks at ≈613–623 cm^−1^ correlate with Si–O rocking^31^ or with the silicon substrate.^32^ No evidence for SiNO peak (expected to be at 3375 cm^−1^) following activation and exposure to air was detected. Likewise, the results did not indicate any Si–O–Si peaks expected to be at 1240 cm^−1^.^28^ It should be noted that the signal-collecting depth in the ATR measurements is a few micrometers, hence the measured spectra reflected not only the surface but also some bulk properties.

AFM measurements were performed to characterize the surface roughness prior to and following plasma activation. The surface roughness was measured in five 2 μme× 2 μm locations on the wafer. The root mean square (RMS) roughness of the glass and the SiN surface is given prior to and after activation in Table 3. Prior to activation the RMS roughness of the glass was approximately four times higher than that of the silicon nitride grown on polished silicon. Activation by nitrogen seemed to slightly increase the roughness both on glass and on silicon nitride, whereas activation by oxygen plasma had no effect on the surface roughness. In general oxygen plasma is more reactive than nitrogen plasma, hence one could expect the opposite. However, this result seems to be in line with the XPS measurements showing that the depletion of substrate nitrogen was larger when using nitrogen plasma than upon using oxygen plasma. It might be that this larger depletion with nitrogen is specific and stemmed from strong affinity between excited atomic nitrogen in the plasma and substrate nitrogen.

**Fig. 10:**
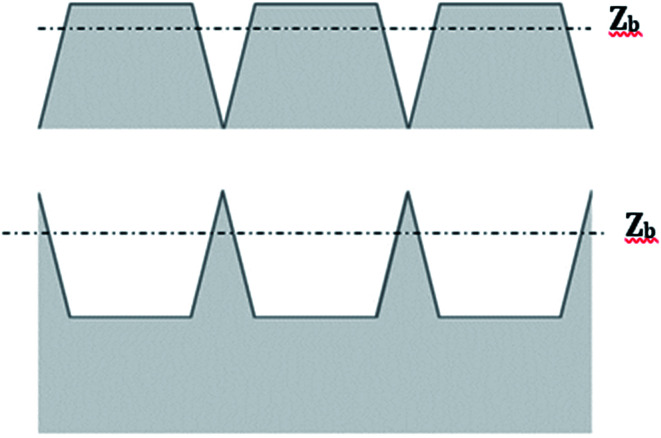
Two surface profiles having identical RMS roughness, but different bearing ratio.

The RMS roughness is often considered as a predictor for the expected quality of a bond. For example, a value higher than 1 nm is regarded as the highest limit for direct bonding.^14^ However, as shown in Fig. 10, the same RMS value might indicate different contact areas. Therefore, cautious should be taken when relying on this parameter.

According to N. Miki *et al.*,^33^ when the surface chemistry is similar, the bond strength is proportional to the actual area of contact. The actual area of contact is defined as the area close enough to the interface so that it feels the attractive forces between the two bonded surfaces (mostly hydrogen bonding forces in our case of direct bonding of hydrophilic surfaces). The distance at which the attractive forces are felt is called the bearing depth (*Z*_b_). The bearing depth is a property of the type of surface, reflecting the material from which the surface is made and the type of interactions involved in bonding. Defining the plane connecting the highest points of a surface as zero and *Z*_b_ as the bearing depth, the probability of bonding two given materials is proportional to the bearing ratio defined as the fraction of area residing less than a distance *Z*_b_ from the zero plane. Considering the thickness of a water layer found at the interface between two hydrogen-bonded surfaces, the bearing depth for silicon is often taken as 1.4 nm.^33^

Taking *Z*_b_ to be 1.4 nanometer, the AFM images were used to calculate the bearing ratios prior to and after activation (Table 4). The bearing ratio of glass was found to increase upon activation, from 0.67 to 0.81 and 0.93 for activation with oxygen plasma and nitrogen plasma, respectively. These findings are in line with the stronger binding between surfaces that were activated with nitrogen in comparison with surfaces that were activated by oxygen plasma. For SiN surfaces, the bearing ratio was maximal regardless of treatment, due to the superb smoothness of the raw material, which was not considerably damaged upon activation.

**Table 4:** The RMS roughness and the bearing ratios of glass and SiN prior to and following activation

	RMS roughness (nm)			Bearing ratio (%)		
	Prior to activation	Following activation with O_2_ plasma	Following activation with N_2_ plasma	Prior to activation	Following activation with O_2_ plasma	Following activation with N_2_ plasma
Glass surface	0.39	0.38	0.5	67	81	93
SiN surface	0.11	0.12	0.18	100	100	100

Advancing water contact angles measurements of the surfaces of glass and SiN were performed prior to and after activation. Glass shows very high hydrophilicity already prior to activation. Therefore, the change in wettability upon activation was almost nil (from 2° for a well-cleaned surface to 1°). In contrast, SiN is less hydrophilic (an averaged advancing water contact angle of 24° prior to activation). Upon activation and exposure to air the water contact angle dropped to 2° regardless of activating gas, indicating that the surface became highly hydrophilic, alas slightly less hydrophilic than the activated glass.

To better identify whether there were any differences between the glass and the SiN surfaces after activation, both surfaces were covered with an ultrathin layer of hexamethyldisilazane (HMDS, (CH_3_)_3_–Si–NH–Si(CH_3_)_3_), known to be attached to oxides and hydroxides surfaces, thus altering the surface into hydrophobic. HMDS binds to water-free surfaces *via* its silicon atoms that form chemical bonds with surface oxygens, while being cleaved, thus releasing a molecule of ammonia. Therefore, high water contact angle with HMDS indicates high hydrophilicity prior to its coating. A clear difference was observed between glass-coated surfaces and SiN-coated surfaces. The advancing water contact angle of HMDS-coated glass was 82° when the coating was performed on non-activated glass, and 75° when the coating was performed on activated surface (regardless of gas type). For SiN, the advancing water contact angle of HMDS-coated surface was 66° when the coating was performed on non-activated SiN and 57° when the coating was performed on activated surface (again, regardless of gas type). Regardless of treatment, the contact angles for HMDS on glass were always higher than for HMDS on SiN. This proves that the glass surface is more hydrophilic than SiN not only prior to activation, but also following activation. Unexpectedly, the water contact angle of HMDS coated on non-activated surfaces was higher than the water contact angle of HMDS coated on activated surfaces. This rather surprising result is explained by the fact that the coating process was performed immediately after activation, such that the activated surfaces was not exposed sufficient time to adsorb humidity from the air (in contrast to the introduction of the bonded wafers to DI water prior to bonding). This explanation is in line with common knowledge on the need for humidity in air in order to obtain high quality silicone-based self-assembled monolayers.^34^

## Discussion

This work has demonstrated for the first time that SiN can be bonded to glass by a direct bonding procedure, based on activating both surfaces with plasma. The bonded pairs were characterized by examining voids quantity and area, shear strength measurements and by the crack opening method. Various parameters of the bonding process were altered across the experiments to study their influence on the bond quality. Comparing the mechanical properties of the bonded samples as a function of the operational parameters that govern activation revealed that samples that were activated by nitrogen plasma and annealed at 400 °C had superior mechanical properties relative to samples activated under other conditions. While this observation was found to be quite solid, repeatability remained a challenge. The success in bonding SiN to glass raises a question about the possibility of using direct bonding to bond two SiN surfaces. Here, bonding two silicon nitride wafers, using the best parameters found for the glass–SiN direct bonding, was found to be unsuccessful, thus suggesting that the bonding between SiN and glass is at large due to specific conditions prevailing on the surface of the activated glass prior to bonding.

### The effect of activation on glass

The activation of glass by plasma is manifested by surface changes that are both physical and chemical. From the physical point of view the bearing ratio of glass, calculated from the AFM results, increased for both types of activating gases. From the chemical point of view the major effect of activation was found to be the formation of silanol group (Si–OH) on the surface, as noticed by ATR-FTIR measurements and supported by the XPS measurements. The formation of silanols is attributed not only to the activation process by itself but to the exposure of the activated wafers to air and to its humidity. This claim is based on the large number of silanols found also in the N_2_-activated wafers, which seems (albeit not proved) to be larger in the N_2_-activated wafers than in the O_2_-activated wafers. The XPS results, showing an increase in the O : Si atomic ratio following activation and exposure to air, support this notion, as the excess of oxygen is likely to come from the environment (O_2_ in air or humidity) rather than the glass bulk.

The formation of a hydroxylated glass surface hardly had any impact on the water contact angle of the glass surfaces, as the surface was already highly hydrophilic. This pre-activation hydrophilicity (which was higher than that usually observed with clean silica) might be attributed to the presence of the ions in the glass, which are known to increase the number of non-bridging oxygens. These non-bridging oxygens are easily hydroxylated, thus decreasing the water contact angle.^35,36^

### The effect of activation on SiN

The XPS measurements clearly showed the appearance of oxygen on the surface of SiN upon activation. This oxygen was attributed to the formation of oxide on the surface. Quite interestingly, the XPS results showed an increase in the Si : N ratio at the surface. The source of this increase, which may suggest some disappearance of nitrogen, is not fully understood. It is noteworthy that HR-TEM EDS measurements of the interface of bonded samples corroborated the disappearance of some nitrogen close to the bonded surface of the SiN. The oxidation of silicon nitride did not lead to the formation of surface hydroxyls as was inferred from the ATR FTIR measurements. The water contact angle on activated SiN was reduced abruptly reflecting the formation of surface oxide. Still, the presence of small amount of surface hydroxyls cannot be ruled out. This conclusion is supported by the lower contact angle measured following treatment of the activated surface with HMDS in comparison with glass.

### The bonding between glass and SiN

HRTEM imagining of thin films made by FIB from bonded samples revealed a clear boundary between SiN and glass, as expected from non-penetrating surface interactions. EDS profiling revealed an interface no more than 4–6 nm. The interface was characterized by the presence of both silicon, nitrogen and oxygen forming oxynitride as inferred from the EELS measurements.

Overall, and beyond statistical error, samples prepared by nitrogen plasma activation showed superiority over samples prepared by oxygen plasma activation. This superiority was observed regardless of the technique used to assess the mechanical properties of the bonded samples. From the chemical point of view, more silanols were observed on glass upon activation with nitrogen than with oxygen. In parallel, XPS measurements revealed higher O : Si atomic ratio in glass and in SiN treated by nitrogen plasma than in surfaces treated with plasma of oxygen. The fact that the higher ratio was obtained with samples that had been activated with nitrogen plasma testifies for the importance of the post activation conditions (*i.e.* exposure to air and to its humidity) prior to contact, in producing high quality bonding. The larger bearing ratio in glass obtained following nitrogen activation may also contribute to the superiority of nitrogen plasma activation over oxygen plasma activation.

### The mechanism

Based on the described-above findings it can be concluded that for direct bonding between SiN and glass to occur both surfaces have to be activated. Unlike silica–silica bonding (alternatively, bonding between surface-oxidized silicon wafers), where silanol groups appear on the surfaces of in both substrates, the bonding between glass and SiN is asymmetric in the sense that the glass surface contains silanols whereas the amount of silanols formed on SiN is minute. For this reason, the direct bonding between two SiN wafers is very problematic despite their superb smoothness.

Although the activated glass is responsible for supplying the required silanols, the activation of SiN is no less important. Our results suggest that activating the SiN surface is important in order to produce an oxide on the surface that is essential for bonding. The fact that for two silicon wafers having a surface oxide, it is sufficient to activate one wafer only in order to bond the wafers seems to support this explanation.

**Fig. 11:**
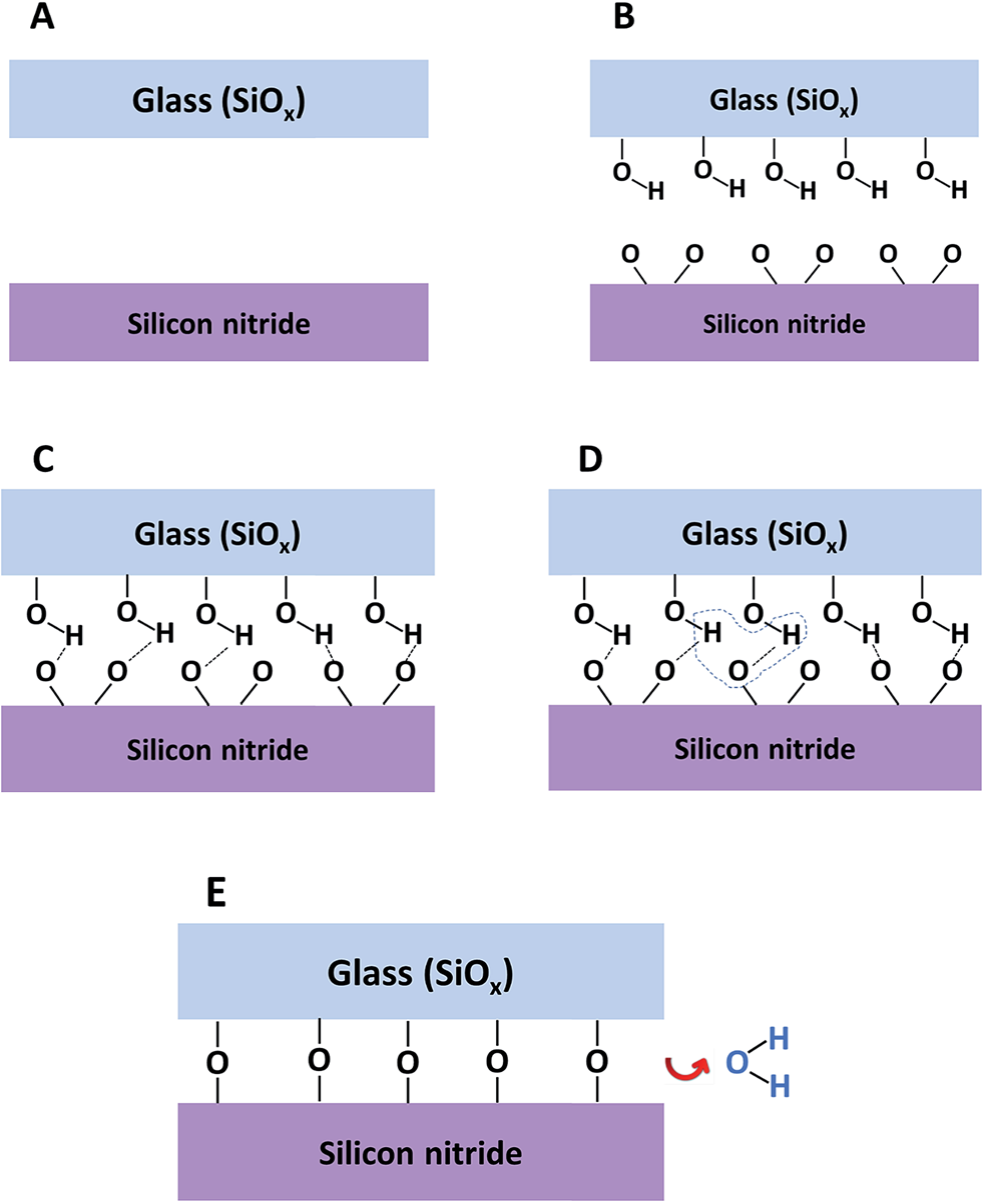
Suggested mechanism for direct bonding of SiN to glass: (A) raw materials, (B) surfaces after plasma activation and exposure to air, (C) surfaces in contact, (D) formation of water molecules, (E) covalent bond formation and water removal.

The mechanism for direct bonding between SiN and glass is presented in Fig. 11. The starting point includes clean, flat and smooth wafers. Activating the wafers with plasma followed by exposure to air causes the formation of –OH groups on the surface of the glass and oxidation of the silicon nitride surface together with some loss of surface nitrogen. Bringing the wafers into intimate contact forms weak and reversible hydrogen bonds between the silicon nitride and the glass surfaces (Fig. 11C). In order to permanently bond the wafers, covalent siloxane bonds between the surfaces must be formed. The covalent bonds are obtained by a condensation reaction (Fig. 11D) during the annealing step. During this step, the water molecules out-diffuse from the interface, dissolve into one of the materials or react with the surfaces and increase the amount of silanol groups. When all the water molecules are removed from the interface between the wafers, strong Si–O–Si covalent bonds are formed (Fig. 11E)

## Conclusions

Direct bonding between silicon nitride and glass substrates was performed successfully for the first time, using low temperature plasma-assisted direct bonding. A direct bonding mechanism between glass and silicon nitride wafers was suggested based on XPS, ATR FTIR, contact angle and HRTEM results. It seems that exposure of the activated surfaces to air and to its humidity is a crucial step for high quality bonding between glass and SiN as it helps to form silanols on the glass and to oxidize the silicon nitride. The observation that activation with plasma of nitrogen may lead to better bonding than activation by plasma of oxygen is in corroboration with this conclusion.

## Conflicts of interest

There are no conflicts to declare.

## Supplementary Material

## References

[cit1] Mirza A. R., Ayon A. A. (1999). Silicon wafer bonding for MEMS manufacturing. Solid State Technol..

[cit2] Tan C. S., Reif R. (2005). Microelectronics Thin Film Handling and Transfer Using Low-Temperature Wafer Bonding. Electrochem. Solid-State Lett..

[cit3] Wada H., Sasaki H., Kamijoh T. (1999). Wafer bonding technology for optoelectronic integrated devices. Solid-State Electron..

[cit4] Lee B., Seok S., Chun K. (2003). A study on wafer level vacuum packaging for MEMS devices. J. Micromech. Microeng..

[cit5] Kim W. K. W. , Wang Q. W. Q., Jung K. J. K., Hwang J. H. J. and Moon C. M. C., Application of Au-Sn eutectic bonding in hermetic RF MEMS wafer level packaging, 9th Int. Symp. Adv. Packag. Mater. Process. Prop. Interfaces (IEEE Cat. No. 04TH8742). 2004 Proceedings, 2004, vol. 35, pp. 215–219

[cit6] Knechtel R. (2005). Glass frit bonding: An universal technology for wafer level encapsulation and packaging. Microsyst. Technol..

[cit7] Suni T. (2006). Direct wafer bonding for MEMS and microelectronics. VTT Publ..

[cit8] Gosele U. (1999). *et al.* Wafer bonding for microsystems technologies. Sens. Actuators, A.

[cit9] Suga T. , Kim T. H. and Howlader M. M. R.. Combined process for wafer direct bonding by means of the surface activation method, 2004 Proceedings. 54th Electron. Components Technol. Conf. (IEEE Cat. No. 04CH37546), 2004, vol. 1

[cit10] Christiansen B. S. H., Singh R., Go U. (2006). Wafer Direct Bonding: From Advanced Substrate Engineering to Future Applications in Micro/Nanoelectronics. Proc. IEEE.

[cit11] Moriceau H. , Rieutord F., Morales C., Sartori S. and Charvet A. M., in Semiconductor Wafer Bonding: Science, Technology and Applications VIII, ed. K. D. Hobart, S. Bengtsson, H. Baumgart, T. Suga and C. E. Hunt, 2005, pp. 34–49, The Electrochemical Society Processings Volume 2005–2006

[cit12] Suni T., Henttinen K., Suni I. (2002). Effects of Plasma Activation on Hydrophilic Bonding of Si. J. Electrochem. Soc..

[cit13] Farrens S. N. (1995). Chemical Free Room Temperature Wafer To Wafer Direct Bonding. J. Electrochem. Soc..

[cit14] Ma X., Liu W., Song Z., Li W., Lin C. (2007). Void-free low-temperature silicon direct-bonding technique using plasma activation. J. Vac. Sci. Technol., B: Microelectron. Nanometer Struct..

[cit15] Aberle A. G., Hezel R. (1997). Progress in Low-temperature Surface Passivation of Silicon Solar Cells using Remote-plasma Silicon Nitride. Prog. Photovoltaics.

[cit16] Martini T., Steinkirchner J., Gosele U. (1997). The Crack Opening Method in Silicon Wafer Bonding. J. Electrochem. Soc..

[cit17] Howlader M. R., Itoh H., Suga T., Kim M. (2006). Sequential Plasma Activated Process for Silicon Direct Bonding. ECS Trans..

[cit18] Gu H., Cannon R. M., Rühle M. (1998). Composition and chemical width of ultrathin amorphous films at grain boundaries in silicon nitride. J. Mater. Res..

[cit19] Muller D. A. (1999). *et al.* The electronic structure at the atomic scale of ultrathin gate oxides. Nature.

[cit20] Harp G. R., Han Z. L., Tonner B. P. (1990). Spatially-Resolved X-Ray Absorption Near-Edge Spectroscopy of Silicon in Thin Silicon-Oxide Films. Phys. Scr., T.

[cit21] Kimoto K., Kobayashi K., Aoyama T., Mitsui Y. (1999). Analyses of composition and chemical shift of silicon oxynitride film using energy-filtering transmission electron microscope based spatially resolved electron energy loss spectroscopy. Micron.

[cit22] Isham M. A. (1992). Technical Paper Gibbs Free Energy of Reactions Involving H20 as a Function
of Temperature and Pressure. NASA Tech. Pap..

[cit23] Fegley M. B. (1981). The Thermodynamic Properties of Silicon Oxynitride. J. Am. Ceram. Soc..

[cit24] Tielsch B. J., Fulghum J. E. (1996). Differential charging in XPS. Part I: demonstration of lateral charging in a bulk insulator using imaging XPS. Surf. Interface Anal..

[cit25] Pereira J. (2009). *et al. In situ* X-ray photoelectron spectroscopy analysis of SiO_*x*_F_*y*_ passivation layer obtained in a SF_6_/O_2_ cryoetching process. Appl. Phys. Lett..

[cit26] Innocenzi P. (2003). Infrared spectroscopy of sol–gel derived silica-based films: A spectra-microstructure overview. J. Non-Cryst. Solids.

[cit27] Efimov A. M., Pogareva V. G. (2006). IR absorption spectra of vitreous silica and silicate glasses: The nature of bands in the 1300 to 5000 cm^−1^ region. Chem. Geol..

[cit28] Tolstoy V. P. , Chernyshova I. V. and Skryshevsky V. A., Handbook of Infrared Spectroscopy of Ultrathin Films, John Wiley & Sons, Inc., 2003

[cit29] Scardera G., Puzzer T., Conibeer G., Green M. A. (2008). Fourier transform infrared spectroscopy of annealed silicon-rich silicon nitride thin films. J. Appl. Phys..

[cit30] Wang J., Liu Q. (2006). Mesoporous silicon oxynitride thin films. Chem. Commun..

[cit31] Scopel W. L., Fantini M. C. A., Alayo M. I., Pereyra I. (2002). Local order structure of a-SiO_*x*_N_*y*_:H grown by PECVD. Braz. J. Phys..

[cit32] Kundu P., Ghosh A., Das S., Bhattacharyya T. K. (2012). Compatibility study of thin passivation layers with hydrazine for silicon-based MEMS microthruster. J. Phys. D: Appl. Phys..

[cit33] Miki N., Spearing S. M. (2003). Effect of nanoscale surface roughness on the bonding energy of direct-bonded silicon wafers. J. Appl. Phys..

[cit34] Wei J., Jianbin L. U. O., Shizhu W. E. N. (2001). Tribological properties of OTS self-assembled monolayers. Chin. Sci. Bull..

[cit35] Zhao P., Kroeker S., Stebbins J. F. (2000). Non-bridging oxygen sites in barium borosilicate glasses: Results from 11B and 17O NMR. J. Non-Cryst. Solids.

[cit36] Rignanese G.-M., Charlier J.-C., Gonze X. (2004). First-principles molecular-dynamics investigation of the hydration mechanisms of the (0001) α-quartz surface. Phys. Chem. Chem. Phys..

[cit37] Karcher R., Ley L., Johnson R. L. (1984). Electronic structure of hydrogenated and unhydrogenated amorphous SiN_*x*_ (0 < *x* < 1.6): A photoemission study. Phys. Rev. B: Condens. Matter Mater. Phys..

[cit38] Hegde R. I., Tobin P. J., Reid K. G., Maiti B., Ajura S. A. (1995). Growth and surface chemistry of oxynitride gate dielectric using nitric oxide. Appl. Phys. Lett..

